# Valuable Lessons for Pharmacist PBRNs: Insights and Experiences from Physician PBRN Members

**DOI:** 10.3390/pharmacy7030123

**Published:** 2019-08-27

**Authors:** Lourdes G. Planas, Shane P. Desselle, Kaitlyn Cao

**Affiliations:** 1 Department of Clinical and Administrative Sciences, College of Pharmacy, University of Oklahoma Health Sciences Center, Oklahoma City, OK 73117, USA; 2 Department of Social, Behavioral and Administrative Sciences, California College of Pharmacy, Touro University, Vallejo, CA 94952, USA; 3 Walgreens, Irving, TX 75063, USA; 4 College of Pharmacy, University of Oklahoma Health Sciences Center, Oklahoma City, OK 73117, USA

**Keywords:** qualitative, interviews, physicians, pharmacists, practice-based research network

## Abstract

Practice-based research networks (PBRNs) rely on a cadre of engaged members to participate in research projects. As pharmacist PBRNs increase in number, it is helpful to understand how members of other professions view their own participation in PBRNs and potential collaborative research endeavors with pharmacists. Due to their longer history of PBRN experience, physician PBRN members may have helpful advice for the establishment of pharmacy networks. The objectives of this study were to describe perceptions among a group of physician PBRN members about: Advice for developing a pharmacist PBRN, practice aspects that might benefit from collaborating with pharmacists who are part of a PBRN, and benefits and challenges of PBRN member participation. This study employed qualitative research methods. Semi-structured interviews were conducted with physician members of the Oklahoma Physicians Resource/Research Network, a primary care PBRN. Advice for establishing a pharmacist PBRN included identifying a champion, recruiting a core group, and conducting a needs assessment. Collaborative areas of interest included medication use management, patient education on chronic disease states, and physician education on new therapies. Participation benefits were categorized as personal satisfaction, improvement in practice quality improvement, advancement of specialty, peer interaction and learning, and real-time information and support. These findings offer insight into strategies for developing and sustaining pharmacist PBRNs and may inform pharmacist PBRN initiatives related to development, member recruitment and retention, and interprofessional project planning with physician PBRNs.

## 1. Introduction

The translation of research findings into practice relies heavily on studies that address problems that are relevant to and intended to improve practice [1]. Practice-based research networks (PBRNs) are critical laboratories for assessing the implementation of evidence-based guidelines into practice [2]. These networks also function as collaborative learning environments to identify, apply, and disseminate new knowledge and solutions to improve patient care processes and outcomes [3]. PBRNs exist in various health professions (e.g., medicine, nursing, dentistry, and pharmacy), as well as in both specialty and primary care [4]. 

The Agency for Healthcare Research and Quality (AHRQ) defines a primary care PBRN as “a group of ambulatory practices devoted principally to the primary care of patients, and affiliated in their mission to investigate questions related to community-based practice and to improve the quality of primary care” [5]. A total of 186 primary care PBRNs are registered with the AHRQ PBRN Resource Center [6]. 

Pharmacist PBRNs have emerged over the past 12 years, largely due to partnerships among pharmacy schools, associations, providers, and researchers. Several notable events ignited PBRN development within the profession of pharmacy. In 2007, the American Association of Colleges of Pharmacy and AHRQ sponsored a national conference titled “Embracing the PBRN Model to Improve Medication Use.” Conference attendees, primarily practitioner–academic pairs from schools of pharmacy, took ideas and insights back to their practice sites and organizations to embark on developing pharmacist PBRNs. The following year, the *Journal of the American Pharmacists Association* (*JAPhA*) published a themed issue on recommended strategies and practitioner–academic collaborations for forming PBRNs [7,8,9]. In 2010, the APhA Foundation published a white paper on “Establishing Pharmacist PBRNs” [10]. 

A search of the AHRQ PBRN Registry and the internet reveals that eight U.S. PBRNs involving pharmacists have been established since 2007. These networks range in geographic coverage, as well as in the backgrounds and practice settings of its pharmacist members. The National Interdisciplinary Primary Care (NIPC) PBRN, composed of 50 family medicine practices with in-house pharmacists in 15 states, studies the team-based care of chronic medical conditions with an emphasis on strategies to improve national guideline adherence [11,12]. The American College of Clinical Pharmacy (ACCP) PBRN, a national network with over 1000 members, is a distinct group of primary care and specialty clinical pharmacists who work in inpatient and outpatient settings [12,13]. Statewide PBRNs of community pharmacists formed over the past decade include the University of Tennessee Pharmacist PBRN (UT Pharm Net) [14], the Minnesota Pharmacy PBRN [15], the Medication Safety Research Network of Indiana (Rx-SafeNet) [16,17], and the Center for the Advancement of Pharmacy Practice Collaborative Research Network (CAPPNet) in Kentucky [18]. Networks with more local coverage include the San Diego Pharmacist Resource and Research Network (SDPharmNet) [19] and the Accountable Care Organization Research Network, Services, and Education (ACORNSEED) in South Florida [20]. 

PBRNs rely on a cadre of engaged members to participate in activities such as peer communication, project idea generation, study patient enrollment, data collection, and practice change implementation. Approximately 13 years after the emergence of pharmacist PBRNs, the number of such networks remains relatively low. Previous survey studies have gathered potential [16,21,22,23] and current [24,25] pharmacist PBRN members’ perceptions regarding interest in joining or participating in a network. Enhanced professional development, the enrichment of patient care, payment for study involvement, and the opportunity to impact community pharmacy and suggest research topics were among the most highly benefits of PBRN participation. Due to their longer history, physician members of established PBRNs may provide valuable insight into developing pharmacist PBRNs, as well as the benefits and challenges of PBRN participation.

In an increasingly complex healthcare environment, attempts to improve patient care should incorporate interprofessional models and solutions. Collaboration between pharmacists and physicians may enhance the impact of practice-based research, especially with regard to patient safety and medication use. Collaborative working relationships between physicians and pharmacists have been studied from the perspective of professional practice [26,27,28,29]. However, little is known about physician views on collaborating with pharmacists on PBRN projects.

The objectives of this study were to describe perceptions among a group of physician PBRN members in the state of Oklahoma about: (1) Advice for developing a pharmacist PBRN, (2) practice aspects that might benefit from collaborating with pharmacists who are part of a PBRN, and (3) benefits and challenges of PBRN member participation. An exploration of physician PBRN member views on these issues may provide insight to development, recruitment, retention, and interprofessional research efforts by current and future pharmacist PBRNs. 

## 2. Materials and Methods 

### 2.1. Study Design

This study employed qualitative research methods. Semi-structured interviews were conducted with study participants, and thematic analyses of the interview transcripts were undertaken to fulfill the study objectives.

### 2.2. Participants

Participants were recruited from physician members of the Oklahoma Physicians Resource/Research Network (OKPRN). Established in 1994, the OKPRN’s mission is to support primary care clinicians through a professional network for peer learning, the sharing of resources for best practices, and practice-based research [30]. Approximately 280 clinicians in 170 practices throughout the state of Oklahoma comprise the OKPRN. Network members have participated in research on a broad array of topics, such as patient–physician email communication [31], patient social support and advocacy [32,33], health information technology and exchanges [34,35,36,37,38], preventive/screening services [39,40,41], hypertension management [42,43], management of lab test results [44], and prescription refills [45].

The OKPRN director invited physician members to participate in the study via the Network’s email listserv. Interested physicians replied, and their contact information was forwarded to the study’s principal investigator to seek informed consent and schedule interviews. Participants selected either a telephone or a face-to-face interview. A convenience sample of 15 physician members constituted the study sample; this number was selected based on the study budget allocation to pay participants for their time in the study (approximately 30 min). The study was approved by the University of Oklahoma Health Sciences Center Institutional Review Board. 

### 2.3. Data Collection

The principal investigator conducted a semi-structured interview with each participant. A semi-structured interview contains a pre-determined set of open-ended questions yet allows the interviewer to ask probing questions as a means of follow-up. This type of interview is helpful to prompt discussion and further explore particular responses. An interview guide containing questions was used (Appendix A). Interview questions were selected based on the study objectives and were reviewed by the OKPRN director and coordinator. Probes and follow-up questions were asked when necessary to maximize quality and depth of participant responses. For example, probes regarding pharmacist–physician collaboration were added based on pharmacy practice research topics. Interviews were audio-taped and transcribed by a third party clerical assistant with prior experience as a transcriptionist for research purposes. 

### 2.4. Thematic Analysis

The thematic analysis consisted of familiarization with the data, generating initial codes, searching for themes, reviewing themes, defining and naming themes, and producing a summary report [46]. The principal investigator and a co-investigator compared the interview transcripts against the audio-recordings for accuracy. They independently generated initial codes from the interview transcripts using NVivo qualitative data analysis software [47]. After an iterative process of consulting with one another on several ocassions, the investigators reached consensus on the initial codes and sorted them into themes. A review of the themes and coding was conducted with the assistance of the third investigator, at which time themes were refined. 

## 3. Results

### 3.1. Study Participants

Most of the participants were male (87%) and white/Caucasian (73%). The median age and number of years in the OKPRN were 51 and 11, respectively. Participants were from a variety of practice types located primarily in central and northeast Oklahoma. Two-thirds (67%) were employees of either a healthcare organization or an academic setting. Four study participants were interviewed in person, and 11 were interviewed by telephone. 

### 3.2. Advice for Establishing a Pharmacist PBRN

Identifying a pharmacist champion who is perceived as a credible leader was seen as an essential activity. Several participants identified the OKPRN director as their Network’s champion and stated that he had visited them in person to discuss joining. Building a core group of members with a strong interest in practice-based research and improving pharmacy practice quality was also perceived as critical. Participants recommended partnering with the state pharmacy association for member recruitment and communication about practice-based research projects. Participants #7 and #9 suggested polling OKPRN members to recommend pharmacists in their areas with whom they would like to collaborate on future PBRN projects.

Striking and maintaining member interest, as well as achieving a balance of members based on practice locations and settings (i.e., rural, suburban, urban), were perceived as important. Developing projects that would be of interest to members and creating a means of disseminating information to them were mentioned. Participant #15 stated, “I think it would be important to have clear goals with, at least at the onset, some simple projects that you could achieve success with fairly simply and build your basis of support.” 

Finally, implementing a planning mechanism, including conducting an initial needs assessment of pharmacists, was advised. A needs assessment could provide information not only about practice-based research topics but also about what incentives to participate are most appealing. Incentives that these participants mentioned experiencing included payment for study participation, the convenience of attending the Network’s annual convocation at the annual meeting of the state’s medical association, and resources such as practice enhancement facilitators to aid with innovation implementation. Table 1 contains a list of participant exemplar quotes related to the advice categories.

### 3.3. Areas for Practice-Based Research Collaboration with Pharmacists

Physician participants discussed areas of their practices that they believed would benefit from collaborating with pharmacists who were part of a PBRN. They mentioned several aspects of medication use, such as improving patient adherence, decreasing medication errors, reducing polypharmacy among older adults, reducing costs and streamlining medication therapies by recommending generic substitutions and combination formulations, and facilitating medication management among patients with chronic conditions (e.g., diabetes, congestive heart failure, and chronic obstructive pulmonary disease). Several physicians noted the benefit of pharmacists as “academic detailers” to educate them on new medication therapies. Some also mentioned the benefits of pharmacists educating their patients about chronic disease states, smoking cessation, asthma inhaler use, and glucometers. Collaboration in terms of planning and coordinating influenza immunizations also was perceived as beneficial. A list of exemplar participant quotes related to these collaboration areas is provided in Table 2.

Many of the physicians preferred collaborating with a consistent pharmacist or pharmacy. Participant #6 practices in a building that also has a pharmacy. He commented, “I’m fortunate that I have an in-house pharmacy on site here. They do diabetic education and training, so I can provide referrals for education and immunizations and it works out excellent.” In contrast, Participant #1 lamented, “It’s harder here because of dealing with chain pharmacies. You basically don’t have any constant figure all the time. It’s who’s on duty for today. When you have a changing population of pharmacists, that really creates a barrier.”

A positive attitude toward working together as a team was evident in some of the participants’ comments. For example, Participant #7 stated, “I work with a pharmacy in [town name] and we co-manage patients.” Participant #13 remarked, “A lot goes on with my patients in the community that I’m not always really aware of, and so I think the more closely we can work together, the better we can both serve our patients.” This sentiment was also expressed by Participant #9: “I believe that my knowledge goes up to a certain point and then after that, I have to depend on those experts. And when I’m dealing with medications, that has to be a pharmacist.”

### 3.4. PBRN Participation Benefits and Challenges

Several themes emerged related to how the participants described benefits and challenges of their PBRN participation. Benefits were categorized as personal satisfaction, improvement in practice quality improvement, advancement of specialty (family medicine), peer interaction and learning, and real-time information and support. Challenges described by participants were categorized as time investment, project funding, staying connected with the PBRN, and organizational support. A list of exemplar participant quotes related to each theme is provided in Table 3.

With regard to real-time information and support, these benefits were primarily accomplished through the Network’s email listserv, which permitted members to ask fellow Network colleagues patient care questions and receive quick responses. Other uses of the listserv included medical literature updates, Network study results, descriptions of best practices, and weekly influenza updates obtained through the State Health Department during flu season. Members also valued testing state-of-the-art health information technology in various studies.

Interacting with and learning from peers, especially from more experienced peers, was viewed as beneficial. As one participant stated, “What you get is not the ‘book’ knowledge, but the knowledge of experience.” Participants in rural and academic settings noted that interacting with their peers helped alleviate feeling isolated from their colleagues. The Network’s annual convocation was mentioned as a valuable forum for socializing, professional interactions, and learning.

Practice quality improvement was observed as an advantage of PBRN participation. By being involved in research, participants were able to adapt their practices, receive feedback to improve, and see meaningful results for their patients. PBRN participation also kept them up to date with relevant topics.

Beyond improvements in their own practices, participants viewed advancing their specialty of family medicine as beneficial. Participants remarked how family medicine struggles for recognition and parity in billing with other medical specialties. Having multiple family medicine practices in the Network involved in research was seen as lending credence to the specialty, strengthening professional identity, and reaffirming that there is power in numbers.

Participants commented that they experienced personal satisfaction through PBRN participation. This satisfaction manifested as feeling excited about research involvement, energized by learning, and assured as a health care provider through the dissemination of study results and receiving feedback from peers on patient care questions.

PBRN participation challenges described by the physicians were categorized as time investment, project funding, staying connected, and organizational support. Time investment challenges included wishing they had time to participate in more projects and finding time to read listserv emails. Examples of project funding challenges were the need for improved contracted health services such as preventative care, recognition from funding sources that would lead to more project funding, and reimbursement for project involvement. Some physicians experienced challenges staying connected with the OKPRN due to rural isolation or distance from the Network’s administration in Oklahoma City. Other challenges experienced by these physicians included gaining approval from their employer groups to participate in Network projects and Institutional Review Board approval issues stemming from their practice settings’ organizational structures.

## 4. Discussion

Perspectives from 15 physician members of a primary care PBRN in Oklahoma were described. Topics included advice for developing a pharmacist PBRN, practice aspects that might benefit from collaborating with pharmacists who are part of a PBRN, and benefits and challenges of PBRN participation. 

When sharing lessons learned from its initial years as a pharmacist PBRN, researchers and network leadership from Rx-SafeNet noted the benefits of conducting “pre-launch” surveys to assess perspectives from potential PBRN members and developing project topics that were relevant to members [17]. This endeavor is similar to the advice provided by the physician participants in the present study to conduct a needs assessment of potential pharmacist PBRN members to identify interest in practice-based research topics. However, Snyder and colleagues cautioned about recruiting pharmacist PBRN members based on interest in a specific project because pharmacists might refrain from joining due to lack of interest in early projects and/or might join to participate in a specific project without comprehending that future projects would be different. These authors noted the need “to assess the balance between fostering early excitement for the network by offering specific targeted research projects and avoiding a confirmation bias that could be detrimental to the future of the organization” [17] (p. 4).

The identification of a pharmacist champion and a core group of pharmacists was described as an essential activity to developing a PBRN. These pieces of advice parallel the use of luminaries in Community Pharmacy Enhanced Services Network (CPESN®) USA, a clinically integrated network composed of approximately 2250 community pharmacies in 46 local CPESNs across the U.S. [48,49]. The primary function of these local networks is to deliver enhanced pharmacy services, such as medication synchronization with clinical review, adherence packaging with patient coaching, and home delivery with patient status review, with a commitment to improve patients’ health outcomes and decrease total healthcare costs. CPESN USA supports these local networks through quality improvement and practice transformation initiatives, such as sharing best practices, providing quality reporting through network dashboards, and coaching participating pharmacies [48]. In doing so, CPESN USA shares various qualities of PBRNs. The Academia-CPESN Transformation (ACT) Pharmacy Collaborative, launched in 2019, may foster growth of practice-based research among local CPESNs [50]. This collaborative was formed to facilitate partnerships between schools of pharmacy and local CPESNs to support the development and implementation of community-based pharmacy care delivery. The results of the present study may be applicable to the growth of CPESN local networks and practice-based research endeavors in the ACT Pharmacy Collaborative. 

Identifying mutually relevant problems that physicians and pharmacists face is an important step to find collaborative solutions that may be examined through practice-based research. Researchers at the University of Wisconsin–Madison used a multi-step interview process to bring together dyads of physicians and pharmacists to describe mutually agreed upon problems and to propose collaborative solutions [51]. Similar to the present study, physicians identified managing patients with diabetes, improving medication adherence, and receiving medication updates from pharmacists. Schools of pharmacy and state pharmacy associations interested in conducting practice-based research or forming a PBRN may benefit from approaching local or state physicians, medical associations, and established PBRNs to identify mutual interests. Such actions may dispel assumptions, educate physicians on pharmacists’ roles, build trust, and identify interprofessional interventions to implement, thus generating practice-based evidence. 

The physicians in the present study discussed their experiences and what PBRN participation means to them. Similarly to previous studies of pharmacist PBRN members [16,21,22,23,24,25], these physicians interpreted benefits that were in their own practices and beyond to their specialty. Through direct observation of practice parameters and patients, they perceived PBRN participation to improve their practices. On a broader scale, they expressed pride in knowing they were contributing to the advancement of family medicine practice through their participation in research. The sentiments of these physicians as they strive to elevate the recognition of their specialty parallel efforts within the profession of pharmacy for patient care services payment and provider status. In a 2011 survey of Kentucky pharmacy members of American Pharmacy Services Corporation, a cooperative of independent pharmacies, respondents reported the opportunity to impact community pharmacy as the most important in the decision to participate in a PBRN [21]. Pharmacist PBRNs seeking members should highlight the opportunity to impact the profession as a whole and, in particular, community pharmacies, by their involvement in practice-based research.

One of the PBRN participation challenges mentioned in the present study was lack of contracted preventative health services. Community pharmacy services aimed at medication optimization and lowering total healthcare costs rely on the notion of preventing medication-related problems that can lead to drug-related morbidity and mortality. For example, the costs of monitoring medication adherence and clinical parameters such as blood pressure pale in comparison to hospital admission costs and other costs associated with cardiovascular events. For sake of longevity and survival, developing PBRNs should take sustainable business models into consideration and explore stakeholder relationships that provide compensation to pharmacists for providing patient care services. 

The limitations of this study should be considered when interpreting the findings. The participant sample was small. Female and ethnic minority physicians were underrepresented. The perspectives of the physicians who took part in the study may have reflected PBRN members with higher degrees of involvement than their fellow Network colleagues. However, the objective of this research was to understand these perspectives to inform recruitment and retention practices by existing and developing pharmacist PBRNs. Therefore, a higher degree of engagement among study participants was desirable. The participants were from a single PBRN, and almost three-quarter were from the same region of the state. Individuals from other networks or from another part of Oklahoma may not share the same perspectives, thus potentially limiting the geographical representativeness of the study findings. More extensive studies with participants from other PBRNs may reveal additional findings. The number of interviews was determined by resources rather than thematic saturation. Probing questions pertaining to collaborative topics were used; however, the majority of participants (67%) mentioned at least two of the predetermined topics without probing. Lastly, almost one-third of the interviews were conducted face-to-face, while the remaining ones were conducted over the telephone. Despite potential differences in the dynamics between these two types of interview formats, there were no discernible differences in responses.

## 5. Conclusions

Physician members of a primary care PBRN provided insights into practice areas that might benefit from collaborating with pharmacists who are part of a PBRN, such as optimizing medication use (improving patient adherence and reducing medication errors) and patient education on chronic disease states. These findings offer insight into strategies for developing and sustaining pharmacist PBRNs and may inform pharmacist PBRN initiatives related to development, member recruitment and retention, and project planning. Various opportunities exist to conduct interprofessional research between pharmacist and physician PBRNs.

## Figures and Tables

**Table 1:** Advice for establishing a pharmacist practice-based research network.

Advice Categories	Exemplar Quotes
Champion/Leader	“The first thing that you’re going to have to have is a champion who is known and respected by pharmacists throughout the state.”
Core group	“Form a core network of people that have buy-in, who basically want to improve outcomes, who have good relationships with a few providers.”
Members (generate interest)	“The initial thing is really to enroll people who are very interested. I think that will probably help you have success with it just from their enthusiasm and their use of the network.”
	“The most effective way is to have a personal relationship with that pharmacist and invite them personally.”
Members (balance)	“Making sure that you have a chance to get broad based and that you have a strategy for getting out to the rural areas and making sure there’s contact with the folks at the private small areas, as well as the larger ones that really keep you in balance.”
	“I think you probably need to get folks from the community and maybe throughout Oklahoma, from the small town, maybe the private small pharmacist who runs his or her business and then to maybe some of the corporate, conglomerate, large pharmacies… and that might be interesting to get different perspectives.”
Projects	“I think it would be to have clear goals with at least at the onset some simple projects that you could achieve success with fairly simply and build your basis of support.”
Needs assessment/planning	“Start with a needs assessment of pharmacists throughout Oklahoma, find out what are some of the challenges they are having, you know, the different community settings that they work in, whether it might be relationships with other physicians or job burnout or pay issues… kinda find out what are pharmacists doing… or what kind of goals or likes do they have in terms of making a contribution and see if they might find it very useful and then start off with perhaps some short term goals and long terms goals.”

**Table 2:** Potential pharmacist–physician collaboration areas within a practice-based research network.

Collaboration Area	Exemplar Quotes
Medication use management	“The other thing that would really be useful is a way of knowing how often the patient is taking the medication by looking at their refill behavior and then knowing which medication doesn’t really make sense for them to be on.”“ If we can collaborate with pharmacists to actually ensure that there’s an accurate record and we can minimize errors, then it’s better for patient safety.”“ I think there will be a greater need in the future for pharmacists to work closely together with physicians as we take care of more chronically ill elderly people who require a lot of medications.”
Patient and caregiver education on chronic disease states	“A lot of our patients might take their medication only when they think their blood pressure is elevated or take their diabetes medication when they think their sugar is elevated as opposed to taking it every day. I think those are important aspects that might be looked at from the pharmacy perspective in terms of helping clinicians do better and helping patients do better.” “I think my pediatric population, parents and kids, especially asthmatics, could have very good things come out of a collaboration with a pharmacist, especially when it comes to inhalers.”
Cost management (e.g., product selection, formulations)	“Something that would be very helpful is having a pharmacist involved in pulling all the charts where the patients are on really expensive medications or their meds could be combined or put into an easier format for them where could have some cost savings.”
Physician education on new therapies	“Doctors don’t get a whole lot of education once they get out, and a lot of it is from the drug reps and we always have to kind of take that with a grain of salt. I would love to have a pharmacist come and say, ‘Okay, here’s these three drugs and here’s when you give this one as opposed to this one and this is what you have to watch out for.’ That would be hugely beneficial.”
Immunization coordination	“I think everything from when we need to do mass immunization programs to where there needs to be some coordination between supply of the material, as well as distribution of it to the people who benefit.”

**Table 3:** Thematic summary of practice-based research networks participation benefits.

Theme	Subthemes	Exemplar Quotes
Real-time information and support	Utilize technology resources	“It’s definitely been huge with the monitoring of, and being involved in, the influenza tracking because you could see when it hits in Oklahoma and then you can treat appropriately.”
	Access user-friendly listserv for timely feedback	“… if you have the ability to ask a question and get an answer from experienced people in a very short period of time, that’s a huge benefit.”
	Disseminate information	“I think the concept of sharing information with clinicians throughout Oklahoma and finding a vehicle to… quickly disseminate it was important.”
Peer interaction and learning	Interact with clinicians from different backgrounds	“Probably the biggest benefit is simply being able to see the forum of doctors and all different practice types all across the state talking about different issues of well, ‘How do you address this?’ and ‘How do you address that?’”
	Learn from clinicians with more experience	“What you get is not the ‘book’ knowledge, but the knowledge of experience.”
	Alleviate isolation	“It gives me a little bit more of a feel for what’s happening at the community level. I think in academic medicine we sometimes become isolated and we become siloed in and the OKPRN is a network that allows us to communicate back and forth.”
	Attend annual conferences	“You bring doctors who participated in the network for one day’s worth of CE or maybe a day and a half worth of CE and then you get to exchange, which is again another way of learning how to improve the delivery of care… sometimes you might have physicians that might have figured out how to get things done, so that’s been beneficial.”
Improvement in practice quality	Keep up to date	“Well, as physicians, we need to keep abreast of what is going on in medicine and the best way to do that is to be involved in decision making and research… particularly what it involves with my patients in family medicine.”
	Receive feedback on practice parameters	“I feel I have made some of my office activities more streamlined and more beneficial to [my patients] for quality and access.”
	See patients experience benefits	“… I’ve been involved in so many different projects through the years that have been associated with OKPRN, such that my patients can actually reap the rewards.”
Advancement of family medicine	Participate in research	“The practice stimulation of engaging in clinical research was a big benefit; and then just knowing that we were involved in significant research for the specialty itself was also pretty exciting.”
	Lend credence to specialty	“Being an active member means that we can be in touch with all the good things that are being done there, which has… a lot of significance to what family care practice is all about.”
Personal satisfaction	Excited about research involvement	“I just love all the projects… I’m like a kid in a candy store with that kind of stuff.”
	Energized by learning	“… I think that’s what keeps me invigorated, is learning new stuff all the time.”
	Assured as a health care provider	“It just helps you deliver better care because you’re not second-guessing what you’re doing.”

Abbreviations: OKPRN, Oklahoma Physicians Resource/Research Network; CE, continuing education.

## Appendix A. Interview Guide

1. Why did you decide to join OKPRN?
2. What did you think were the biggest benefits to joining OKPRN?
3. Did you have any initial hesitations or concerns about joining OKPRN?
  - (If yes…) After having been in the Network, do you think your concerns were justified?
  - What additional challenges have you come across as a participant in OKPRN, and how have you dealt with them?
4. How has participation in the Network affected your practice and your patients?
5. What do you think can be done to make OKPRN an even better experience for you and other physicians?
6. We are developing a similar network among pharmacists in Oklahoma. What are your thoughts on a pharmacist network?
7. What aspects of your practice might benefit from collaborating with pharmacists who are part of a network?
8. [Probe if not mentioned]
  - Medication reconciliation
  - Smoking cessation
  - Training and educating patients in self-management, such as diabetes or hypertension
  - Working with special populations (pediatric, geriatric, ethnic minorities)
9. What advice do you have for us in establishing a pharmacist network?
